# Evaluation of orthognathic surgery on articular disc position and temporomandibular joint symptoms in skeletal class II patients: A Magnetic Resonance Imaging study

**DOI:** 10.4317/jced.53824

**Published:** 2017-08-01

**Authors:** Gholamreza Firoozei, Shirin Shahnaseri, Hasan Momeni, Parisa Soltani

**Affiliations:** 1 Senior (retired) Assistant Professor, Department of Oral and Maxillofacial Surgery, School of Dentistry, Isfahan University of Medical Sciences, Isfahan, Iran; 2 Assistant Professor, Dental Implants Research Center, Department of Oral and Maxillofacial Surgery, School of Dentistry, Isfahan University of Medical Sciences, Isfahan, Iran; 3 Assistant Professor, Department of Oral and Maxillofacial Surgery, Islamic Azad University of Isfahan (Khorasgan Branch), Isfahan, Iran; 4 Post-graduate Student, Department of Oral and Maxillofacial Radiology, School of Dentistry, Isfahan University of Medical Sciences, Isfahan, Iran

## Abstract

**Background:**

The purpose of orthognathic surgery is to correct facial deformity and dental malocclusion and to obtain normal orofacial function. However, there are controversies of whether orthognathic surgery might have any negative influence on temporomandibular (TM) joint. The purpose of this study was to evaluate the influence of orthognathic surgery on articular disc position and temporomandibular joint symptoms of skeletal CI II patients by means of magnetic resonance imaging.

**Material and Methods:**

For this purpose, fifteen patients with skeletal CI II malocclusion, aged 19-32 years (mean 23 years), 10 women and 5 men, from the Isfahan Department of Oral and Maxillofacial Surgery were studied. All received LeFort I and bilateral sagittal split osteotomy (BSSO) osteotomies and all patients received pre- and post-surgical orthodontic treatment. Magnetic resonance imaging was performed 1 day preoperatively and 3 month postoperatively. Descriptive statistics and Wilcoxon and Mc-Nemar tests were used for statistical analysis. *P*<0.05 was considered significant.

**Results:**

Disc position ranged between 4.25 and 8.09 prior to surgery (mean=5.74±1.21). After surgery disc position range was 4.36 to 7.40 (mean=5.65±1.06). Statistical analysis proved that although TM disc tended to move anteriorly after BSSO surgery, this difference was not statistically significant (*p* value<0.05).

**Conclusions:**

The findings of the present study revealed that orthognathic surgery does not alter the disc and condyle relationship. Therefore, it has minimal effects on intact and functional TM joint.

** Key words:**Orthognathic surgery, skeletal class 2, magnetic resonance imaging, temporomandibular disc.

## Introduction

An important issue in the orthognathic surgery is its effects on temporomandibular (TM) joint. Determination of condylar position in relation to TM disc is of utmost importance as changes in the condyle-disc complex position can result in complications, malfunction and delayed relapse of orthognathic treatments in achievement of a successful orthognathic surgery, maxillofacial surgeons mainly rely on patient satisfaction from esthetic aspect. However, one of the most important indicators for achieving success in these cases is the functional restitution of the patient. TM joint is the basis of occlusion and direct and indirect interventions in its structure can affect daily life of the patients (1).

 Recording and monitoring of the condylar position and TM joint morphology after orthognathic osteotomy such as Sagittal Split Ramus Osteotomy (SSRO) or Intraoral Vertical Ramus Osteotomy (IVRO) are remarkable. Determination of disc- condyle complex relation is cornerstone of such assessments. However, due to high costs and complexity of available techniques, limited stu-dies have been carried out in this regard (2).

Currently, two methods are available for evaluation of TM disc position and morphology. The first method is arthrography which is performed by fluoroscopy after injection of contrast liquid media in superior and inferior articular spaces. This technique produces an indirect image from the articular disc (2). Another method is Magnetic Resonance Imaging (MRI) in which magnetic field and pulses of radio waves are used instead of ionizing radiation to obtain images. As MRI can produce high quality images of the soft tissue, it can be ideal for radiographic evaluation of the articular disc.

The exact influences of orthognathic surgeries on TM joint morphology and function are not clear yet. Experimental, clinical and radiological studies have been performed to clarify the effects of orthognathic surgery on the relation between the condyle and the disc. For instance Sanroman *et al.* evaluated morphometric and morphologic changes in TM joint following orthognathic surgery using MRI and Computed Tomography (CT) scan. The authors concluded that although changes were observed in bony structures of the TM joint following bimaxillary surgery, these alterations are temporary and do not affect the final results of the surgery (2). Another study including cone-beam CT scanning after orthognathic surgery revealed that in most patients changes in linear and angular position of condyles were inconsiderable (3). Moreover, Lee *et al.* aimed to evaluate the effects of orthognathic surgery for treatment of skeletal class 3 deformity on disc position and TM disorders symptoms using clinical and radiographic examinations and suggested that orthognathic surgery do not alter position of TM disc (4). However, a study carried out on 25 patients requiring orthognathic surgery for correction of skeletal class 3 deformity reported that clinical and MRI examinations revealed correction of articular disc position after surgery (5). Ueki *et al.* concluded that SSRO does not improve anterior disc displacement, IVRO improves anterior disc displacement in initial postsurgical period, and both approaches may improve TM joint symptoms (6). In the study of Mavreas *et al.*, TM tomography showed that condyles were replaced inferior and anteriorly following surgical correction of skeletal deformities. However, the condyle moved to the original position after 6 months (7).

As changes in TM disc position can exacerbate or improve symptoms in the TM joint, the purpose of this study was to determine and compare the position of the articular disc before and after orthognathic surgery in skeletal class 2 patients and to assess whether orthognathic surgery leaded to changes in TM disc position.

## Material and Methods

This observational study was performed on patients referred to Alzahra Medical Center in 2015 requiring orthognathic treatment for correction of skeletal class 2 deformities. 15 patients with skeletal class 2 deformity (based on Steiner and Witts analysis) who underwent fixed orthodontic treatment for an average of 12 months achieving ideal dental arch form were selected for the study. Inclusion criteria were: a) the patient had been treated with fixed orthodontics prior to the surgery, b) the patient had a functional TM joint, c) the patient did not have any developmental syndrome, d) the patient did not have any systemic contraindication for surgery, e) bilateral sagittal split osteotomy (BSSO) for mandible and LeFort 1 for maxilla, and f) the patient had a skeletal class 2 deformity. The study was approved by Isfahan Regional Bioethics Committee (No. 386412). Patients signed the informed consent form prior to the study and were excluded from the study if they were not willing to participate or perform MRI before or after orthognathic surgery.

BSSO osteotomy sites were fixed using three 13 mm screws on each side of the mandible. Moreover, maxillary osteotomy sites were fixed by four miniplates and sixteen 7 mm screws.

MRI was obtained 1 day prior to and 3 months after the surgery and objective and subjective signs of TM joint were evaluated and recorded.

Images of TM joint were obtained using one device (SIGNA Scanner, General Electric, IL, USA) with 1.5 T magnetic field and TM joint coil with two 6.5 cm surfaces. The imaging protocol included gradient echo, T1 weighted, dual echo and multiple echoes.

TM joint images were obtained in closed mouth resting position and then maximum opening. Bilateral TM joint MR images were obtained using following parameters: sagittal, eight to ten 3-mm sections, 10 cm field of view, TR=469 ms, TE=30 ms. Then, the mouth was opened gradually using a biting device which allowed dynamic imaging of the TM joint. These images were acquisitioned using following parameters: sagittal, TR=100 ms, TE= 20 ms. Images were transferred to computer by a scanner device (GT9500, Epson, Tokyo, Japan) for evaluation.

**Figure 1:**
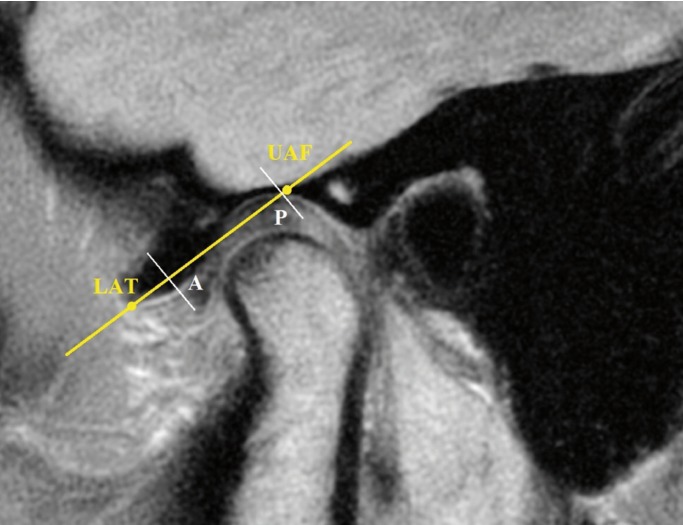
Evaluation of disc position on MR images.

Figure 1 depicts the method for determination of the articular disc position derived from the method described by Ueki *et al.* (6). In order to trace the position of the TM disc, a line was drawn from the uppermost point of the articular fossa (UAF, marked as 10) to the lowermost point of the articular tubercle (LAT, marked as 0). This line was continued anteriorly and inferiorly. If the anterior border of the disc was anterior to this line, it was considered negative. These two points were chosen because they did not change with remodeling.

Perpendicular lines to this line were drawn in the anterior and posterior borders of the disc. Finally, disc position was determined by averaging anterior (point A) and posterior (point P) disc limits. Each MR image was traced three times and the mean value of these three measurements was determined as disc position.

Other variables such as hypermobility (maximum mouth opening more than 50 mm), disc degeneration, bony degeneration, joint sounds and joint tenderness were evaluated using MR images and clinical examinations to assess the health and functional ability of the TM joint.

Statistical Package for the Social Sciences (SPSS, version 22, NY, USA) was used for statistical analysis. For this purpose descriptive statistics, Wilcoxon test, and Mc-Nemar test were used and level of significance was *p*=0.05. MR images were coded to prevent bias.

## Results

**Figure 2:**
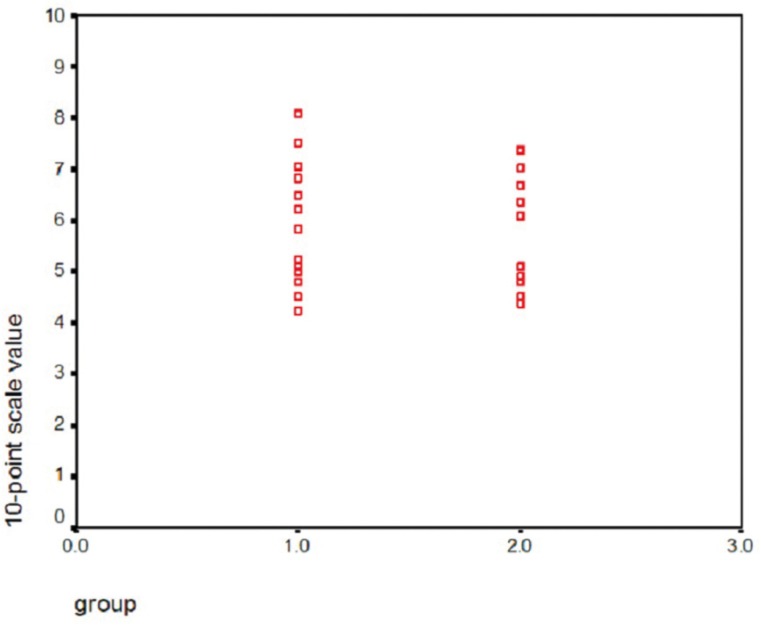
Diagram of disc position relative to the drawn line on a 10-point scale (n=15).

Disc positions before the surgery ranged from 4.25 to 8.09 (mean=5.74±1.21). After the surgery disc positions ranged from 4.36 to 7.40 (mean=5.65±1.06). According to Wilcoxon test, although the articular disc tended to replace anteriorly in patients after BSSO surgery, however, this difference in disc position was not statistically significant (*p*>0.05). Figure 2 shows the diagram for disc position relative to the drawn line on a 10-point scale.

In MRI evaluation of patients before and after the surgery no evidence of erosion of the condylar head or bony degeneration of the glenoid fossa was observed. Hypermobility of the TM joint was diagnosed in three patients based on pre-surgical MR images. This finding was not observed in any of the post-surgical images. However, this difference was not statistically significant.

MRI before surgery showed normal morphology and position of TM disc in relation to the articular fossa and condyle. However, after surgery 5 patients (8 joints) showed evidence of disc deformation and degeneration although the difference between pre- and post-surgical images was not significant in this regard.

Initial click was present in 5 patients (10 joints). However, MRI findings did not indicate any deformation or displacement. These 5 patients also reported tenderness in their masticatory muscles. After surgery, initial click was not present in any of the patients. This difference did not reach to the level of significance.

Masticatory muscles had tenderness preoperatively in 6 patients. Involved muscles included temporalis muscle (n=1), masseter muscle (n=2) and lateral pterygoid (n=3). Tenderness in masticatory muscles was observed in 3 patients as indurations in masseter and temporalis muscles in patients who reported tenderness in these muscles pre-operatively. Muscle tenderness was resolved in other patients after surgery. However, this difference was not statistically significant.

## Discussion

The aim of orthognathic surgery is to correct facial deformities and dental malocclusion and restitution of normal orofacial functions. However, there is still controversy if orthognathic surgery can reduce the TM joint symptoms or may cause TM disorders.

Three possible reaction of TM joint to orthognathic surgery include none, adaptive deformation, and degenerative deformation. In the first condition no alterations occur in TM joint soft and hard tissue. In adaptive deformation, regional reconstruction, maintained ramus height, normal mandibular growth (if remained after surgery), and stability of B point (supramental point) is observed. In degenerative deformation, the whole condyle undergoes remodeling, ramus height is decreased, mandibular growth is hindered, and B point is repositioned (8). These morphologic deformations in bony structures of the TM joint can be assessed by clinical and radiographic examinations. However, articular disc is another part that may undergo these deformations. In fact, some of TM joint dysfunctions are caused by disturbances in disc-condyle complex. Some of these problems are results of changes or relocations in disc and condyle relation. Others are caused by inconsistency between articular surfaces, disc, condyle, and glenoid fossa. The other reason of TM dysfunctions is movements beyond the normal range of motion in relatively normal tissues. Orthognathic surgeries such as SSRO and IVRO can cause changes to TM joint in the aforementioned ways.

Factors contributing in dislocation of condyle during orthognathic surgery include recumbent position of the patient under general anesthesia, method of fixation of osteotomy fragments, surgical techniques, bony interference between proximal and distal fragments, proper manipulation of the proximal fragment during fixation, and changes in occlusal plane (9). A change in condylar position results in changes in disc position. Therefore, in order to prevent TM joint dysfunction, condyles should be placed in the proper relation with the articular disc during orthognathic surgery (10,11).

Although in the present study in all 15 skeletal class 2 patients the disc tended to move anteriorly after orthognathic surgery, however, this difference was not statistically significant. This is consistent with the results of Kim et al. study in which MRI had revealed that disc position in closed mouth did not change following BSSO surgery (12). Moreover, the study of Fang *et al.* in 2009 reported the same findings as disc position did not change significantly after BSSO surgery for correction of skeletal class 3 deformity (13). In another Study, Ueki *et al.* compared the condylar changes in SSRO surgery with and without LeFort I osteotomy. They concluded that TM disc is not displaced following SSRO surgery either with or without LeFort I osteotomy (6).

Freihofer *et al.* in their study including condyle radiography of 38 patients with SSRO for mandibular advancement reported that in 10 cases disc-condyle complex tended to move anteriorly (14). This is coherent with the results of our study in which disc-condyle complex tended to move anteriorly. However, Lee et al. in their study performed on 36 patients with SSRO surgery reported that condyles tend to move posteriorly after SSRO surgery (6). These differences in results of the study of Lee et al. and the findings of the present study and Freihofer *et al.* study indicates the need for further studies including more samples.

Gaggl *et al.* in 1998 evaluated clinical and radiographic findings of TM disc displacement before and after orthognathic surgery in skeletal class 2 patients. Based on their study on 25 patients, disc position improved after the study (5). In their study disc position is considered a qualitative variable and this may reduce the accuracy of their findings. In the present study two stable points are defined in the TM joint which do not undergo remodeling and this can be the reason of inconsistencies between findings of Gaggl *et al.* study and our study.

In 2006 Parrot *et al.* assessed condylar changes following orthognathic surgery using cone-beam CT. Most patients showed inconsiderable changes in linear and angular position of the condyle (3). Similar to our study, this study mentioned that orthognathic surgery has minimal effects in TM joint function and morphology.

In the present study TM joint symptoms were relieved in all patients after orthognathic surgery. However, this did not reach the level of significance. This may be due to small sample size. Ueki *et al.* in their study on 43 patients concluded that TM joint symptoms are improved following IVRO and SSRO surgeries in short-term. Future studies with larger sample size can help to clarify if orthognathic surgeries are effective in revealing TM joint symptoms in patients with skeletal deformities.

## Conclusions

Based on the findings of the present study, orthognathic surgery does not alter condyle-disc complex relations in short-term. Therefore, it has minimal effects in normal and functional TM joint. However, it is unclear if long-term studies represent the same findings.
